# Changes in Components of Metabolic Syndrome after Antiviral Eradication in Hepatitis C Virus Infection

**DOI:** 10.3390/life13020534

**Published:** 2023-02-15

**Authors:** Anca Trifan, Tudor Cuciureanu, Robert Nastasa, Ermina Stratina, Sebastian Zenovia, Cristina Maria Muzica, Laura Huiban, Ana-Maria Singeap, Stefan Chiriac, Catalin Sfarti, Camelia Cojocariu, Irina Girleanu, Horia Minea, Remus Stafie, Adrian Rotaru, Carol Stanciu

**Affiliations:** 1 Department of Gastroenterology, Grigore T. Popa University of Medicine and Pharmacy, 700115 Iasi, Romania; 2 Institute of Gastroenterology and Hepatology, “St. Spiridon” University Hospital, 700111 Iasi, Romania

**Keywords:** chronic hepatitis C infection, metabolic changes, SVR12, direct-acting antivirals therapy

## Abstract

Chronic hepatitis C infection is a systemic disease that affects over 71 million patients all over the world and it is to be considered nowadays as a new cardiometabolic risk factor. This study aimed to evaluate the weight and metabolic changes after viral eradication in patients with hepatitis C virus (HCV) infection. We conducted a prospective study between October 2017 to December 2021, in a tertiary care center, in which we included 132 patients with HCV or cirrhosis. All patients received treatment with direct antivirals (DAAs) and achieved sustained viral response at 12 weeks (SVR12). During the study, clinical laboratory data and Fibroscan examinations were recorded in all patients. The study group was evaluated at the initiation of antiviral treatment, at SVR12, and within an average follow-up period of 6 months to 12 months after the previous evaluation. Evaluation at SVR12 and the data recorded in the post-SVR surveillance period show a further increase in BMI compared with baseline measurements with a statistically significant difference (27.11 ± 3.22 vs. 27.415 ± 3.03 vs. 28.04 ± 1.11 kg/m^2^, *p* = 0.012). The same observation was noticed for waist circumference (WC) at post-SVR evaluation (87.6 ± 13.1 vs. 88.4 ± 13.6 cm, *p* = 0.031). Moreover, the study population registered an increase in the average total cholesterol (TC) values at post-SVR evaluation (177.01 ± 42.2 mg/dL, *p* = 0.014) compared to baseline. In addition, the serum level of triglycerides had been modified after viral clearance, with a minimal decrease in the mean values of triglycerides (TGD) at SVR-12 assessment (133.48 ± 41.8 mg/dL, *p* = 0.78), followed by a significant increase to the mean value of 145.4 ± 47.2 mg/dL (*p* = 0.026) in the third evaluation. Our study highlights that HCV eradication does not improve the lipid profile in the short term, and these patients still have an additional cardiovascular risk factor due to high levels of TC, TGD, and weight gain.

## 1. Introduction

Chronic hepatitis C virus (HCV) infection is still a worldwide health burden affecting an estimated 71 million people globally, with millions newly infected each year, and is a major cause of liver disease, cirrhosis, hepatocellular carcinoma, and death [1]. Due to its high rate of chronicity, HCV involves several metabolic abnormalities, such as metabolic syndrome (MS), obesity, dyslipidemia, diabetes mellitus, and insulin resistance [2].

HCV eradication with Peg-INF/RBV was associated over time with lipid metabolism alterations and lifestyle changes [3]. It has been reported that a decrease in total cholesterol (TC) and low-density lipoproteins (LDL) was seen after interferon therapy. Other researchers reported a post-treatment increase in LDL-cholesterol and TC after antiviral therapy [4]. In the new era of interferon-free antiviral therapy, the behavior of lipoproteins during the treatment and after achieving sustained virological response (SVR) is debatable. It has been reported that alterations in lipid metabolism, such as an increase in LDL-cholesterol and a decrease in triglyceride levels, are observed after interferon-free therapy [5]. Understanding the post-therapeutic changes in metabolic and inflammatory markers is essential given the findings of lipid metabolism and inflammatory response with HCV. After obtaining SVR, more treatment interventions may be required, along with long-term follow-up and care, if metabolic and inflammatory abnormalities still exist [6]. Additionally, this would broaden the scope of long-term HCV patient care encompassing extrahepatic morbidities related to HCV, such as cardiovascular disease and dyslipidemia, in addition to consequences including liver decompensation and hepatocellular cancer. It has been demonstrated that several extrahepatic morbidities, such as diabetes mellitus, are improved by successful HCV elimination [7].

The use of interferon-free regimens has triggered global ongoing concern about the effects of the therapy on lipid metabolism in the long term. Obesity, one major criterion for metabolic syndrome, was a concern for successful SVR. Back in the interferon era, values of body mass index (BMI) ≥ 30 kg/m^2^ represented a negative independent predictor for lack of response to antiviral treatment [8,9]. Despite the SVR, some patients still have steatosis and fibrosis that are clinically significant. People with chronic HCV frequently have liver steatosis, which is seen in 50% to 70% of liver biopsy samples, indicating that HCV directly contributes to the development of steatosis [10]. Cross-sectional and longitudinal studies both support the elevated metabolic risk linked to hepatic steatosis, which is frequently present in people with HCV [11].

A non-invasive method for evaluating liver fibrosis and steatosis is vibration-controlled transient elastography (VCTE) using a controlled attenuation parameter (CAP) [12]. The rapidity, painlessness, and ease of use of this technique, together with its high repeatability and reproducibility, are its advantages compared to liver biopsy. It has also been successfully used in clinical practice [13]. CAP is calculated simultaneously with liver stiffness measurements (LSM) since it utilizes the same ultrasonic signal probe and analyzes the liver’s coefficient of attenuation at a frequency of 3.5 MHz [12]. Several recent studies have discovered a considerable reduction in liver fibrosis after DAA treatment, but on the other hand, the literature contains scant and conflicting information about steatosis [14,15]. This highly efficient therapy with direct-acting antivirals (DAAs) has improved the SVR rate and quality of life, but data regarding changes in BMI and lipoproteins is insufficient and questionable [8,9]. In light of its established link to the development of non-alcoholic fatty liver disease, it is essential for the indirect management of liver disorders to have a better understanding of the metabolic profile of patients beginning DAAs-regimen and the impact of treatment on metabolic variables. However, these studies aimed to assess weight and metabolic changes in chronic HCV patients treated with interferon-free antivirals.

## 2. Material and Methods

### 2.1. Patients

We initially evaluated 430 HCV individuals, but only 132 patients with HCV infection, treatment-experienced or naïve, fulfilled the entry criteria according to the national treatment protocol, and were followed and treated with DAAs for 12 weeks. They all consented to undergo the VCTE examination. From October 2017 to December 2021, the patients were prospectively enrolled at the Gastroenterology and Hepatology Institute in Iasi, Romania. They were recommended to our clinic by general practitioners and other specialists’ coworkers. Patients with other chronic liver diseases, such as hemochromatosis, Wilson’s disease, alcohol consumption up to 20 g/day for women and >30 g/day for men, use of steatogenic medications, pregnancy, cancer, heart failure, and end-stage renal diseases, as well as patients with unreliable VCTE and CAP measurements were all disqualified from the study. Different regimes of DAAs were used (Paritaprevir/Ritonavir/Ombitasvir and Dasabuvir or Sofosbuvir/Ledipasvir).

### 2.2. Clinical and Laboratory Assessment

On the same day, patients underwent a VCTE assessment, a thorough clinical examination, and laboratory testing. The demographic and clinical information collected included information on sex, age, daily alcohol and cigarette use, BMI, waist size, type of diabetic medication, and systolic and diastolic blood pressure. Hemoglobin, the international normalized ratio (INR), fibrinogen, fasting plasma glucose, hemoglobin A1c (HbA1c), alkaline phosphatase (ALP), bilirubin, albumin, total proteins, urea, serum creatinine, total cholesterol, triglycerides, and low-density lipoprotein are all examples of blood markers. Weight in kilograms divided by the square of height in meters was used to compute BMI. BMI values between 25 and 30 kg/m^2^ and BMI greater than 30 kg/m^2^, respectively, were used to characterize overweight and obesity. The surveillance interval was at the initiation of the antiviral treatment, 12 weeks after treatment completion, and at an average follow-up period of 6–12 months after the second evaluation.

Regarding the criteria for prediabetes, we used two entities: impaired fasting glucose (IFG) defined as fasting plasma glucose ranging from 110 to 125 mg/dL, and HbA1c ranging from 5.7% to 6.4% [16]. The American Diabetes Association proposed fasting glucose 126 mg/dL or HbA1c 6.5% as the acceptance criteria for diabetes in persons with prior diagnoses [17].

For the diagnosis of metabolic syndrome, we used the criteria of the National Cholesterol Education Program (NCEP) Adult Treatment Panel III (ATP III). According to the NCEP ATP III definition, metabolic syndrome is present if three or more of the following five criteria are met: a waist circumference greater than 94 cm for men and 88 cm for women, blood pressure greater than 130/85 mmHg, a fasting TG level greater than 150 mg/dL, a fasting HDL level less than 40 mg/dL for men and 50 mg/dL for women, and a fasting blood sugar level greater than 100 mg/dL [18]. The ethics committee of our institute gave its approval after the study was carried out in accordance with the guidelines of the Declaration of Helsinki. Each person signed a written declaration of consent.

### 2.3. FibroScan^®^ 520 Compact Model Measurements

To check the patients who were a part of our inquiry for liver fibrosis and steatosis, we employed the FibroScan^®^ 520 compact model (Echosens, Paris, France) equipped with the M (normal) or XL (obese) probe. Patients were assessed in the supine posture with the right arm fully extended after at least four hours of fasting. As a result, the intercostal window for right lobe liver scanning was improved. First, an M probe with a 3.5 MHz transducer frequency was used for the inspection. Indicators on the machine employed the XL probe (2.5 MHz) if the distance between the skin and the liver capsule was greater than 25 mm. Reliable measurement was defined as having occurred if 10 acquisitions were made with an interquartile range of no more than 30%. The decibel-milliwatt (dB/m) unit of measurement for CAP is a quantitative technique. For CAP values, the thresholds were 248 dB/m for mild steatosis (S1), 268 dB/m for moderate steatosis (S2), and 280 dB/m for severe steatosis (S3) [19]. Regarding LSM examinations, the study used the following values as the cut-offs: 5.6 kPa for mild fibrosis (F1), 7.1 kPa for significant fibrosis (F2), 9.5 kPa for advanced fibrosis (F3), and 12.5 kPa for cirrhosis (F4) [20].

### 2.4. Statistical Analysis

Utilizing SPSS version 24.0, analysis was carried out. While categorical variables were expressed as absolute values and percentages, continuous variables with normal distributions were expressed as mean SD. To compare categorical data, the chi-square test was employed. Using the Student t-test, quantitative variables having a normal distribution were compared. We employed nonparametric techniques for nonnormal data, such as the Mann–Whitney U test, and the Kolmogorov–Smirnov test to determine whether the data distributions were normal. A *p*-value of 0.05 was used to determine the statistical significance of the results.

## 3. Results

There were 132 patients included in the study cohort, 64.4% of whom were female. The mean age was 61.17 ± 9.1 years, the mean BMI was 27.12 ± 3.22 kg/m^2^, and the mean WC was 87.6 ± 14.1 cm. In the analysis of the baseline sample, 25.7% of the participants were obese, 40.2% were overweight, and 51.5% of the participants had arterial hypertension. Additionally, 26.5% of people had metabolic syndrome, 24.2% had T2DM, 20.4% had dyslipidemia, and 32.5% of people had pre-diabetes. According to LSM examinations, 16 (12.1%) HCV patients had significant fibrosis (F2), 42 (31.8%) had advanced fibrosis (F3), and 19 (14.4%) individuals had cirrhosis (F4). CAP measurements revealed that 75 (56.8%) HCV patients had liver steatosis, with a median CAP of 233.27 ± 48.26 dB/m (Table 1).

Regarding the assessment of metabolic profile before and after treatment with DAAs-regimens, there was a non-significant decrease in the levels of glycemia (*p* = 0.064), HbA1c (*p* = 0.058) and HDL-cholesterol (*p* = 0.236), but a significant increase in the levels of total cholesterol (*p* = 0.014), triglycerides (*p* = 0.026) and LDL-cholesterol (*p* = 0.017). After DAAs regimens, ALT and AST presented a decrease in their levels, but without statistical significance (42.5 ± 25.21U/L vs. 35.23 ± 22.13 U/L for ALT, *p* = 0.119 and 36.83 ± 21.54 U/L vs. 30.62 ± 23.61 U/L for AST, *p* = 0.097). On the other hand, GGT decreased to normal levels at the evaluation post-SVR compared to baseline (90.4 ± 72.5 U/L vs. 62.4 ± 46.3 U/L, *p* = 0.038). In evaluating the parameters obtained with VCTE, we observed a significant decrease of LSM values (*p* = 0.023). Additionally, approximately two-thirds of individuals (62.1%) had steatosis after viral eradication, with a significant increase in CAP score (*p* < 0.001) (Table 2).

In an analysis in which we included only the clinical and metabolic profile evolution during DAAs regimens, there was a significant change regarding the BMI values before and after the treatment (*p* = 0.012) (Table 3). Evaluation of BMI at SVR12 showed a slight increase (27.415 ± 3.03 kg/m^2^) and the data recorded in the post-SVR surveillance period showed a further rise of BMI compared to the baseline measurements (27.11 ± 3.22 vs. 28.04 ± 1.11 kg/m^2^). The same observation was noticed for the WC during DAAs regimens (87.6 ± 13.1 vs. 87.9 ± 13.2 vs. 88.4 ± 13.6 cm, *p* = 0.031). Additionally, achieving SVR has shown some serious modifications in the lipid profile. The behavior of serum lipid levels differed from the baseline to the post-SVR assessment period. We have observed an increase in TC at SVR and at the post-SVR evaluation (161.68 ± 31.6 vs. 168.23 ± 37.1 vs. 177.01 ± 42.2 mg/dL, *p* = 0.014). In addition, a remarkable increase was seen in the LDL-cholesterol values during the follow-up periods. Compared to baseline, we had a statistical increase in the LDL-cholesterol values (114.05 ± 34.9 mg/dL vs. 122.41 ± 34.3 mg/dL vs. 129. 32 ± 44.6; *p* = 0.07) at post-SVR evaluation. Regarding the levels of triglycerides, the quantitative evaluation at baseline showed an average of 135.7 ± 48.4 mg/dL. At the time of the SVR-12 assessment, there was a minimal reduction in mean values (133.48 ± 41.8 mg/dL), followed by a significant increase of 145.4 ± 47.2 mg/dl found at the third evaluation with a significant statistical difference (*p* = 0.026).

**Figure 1:**
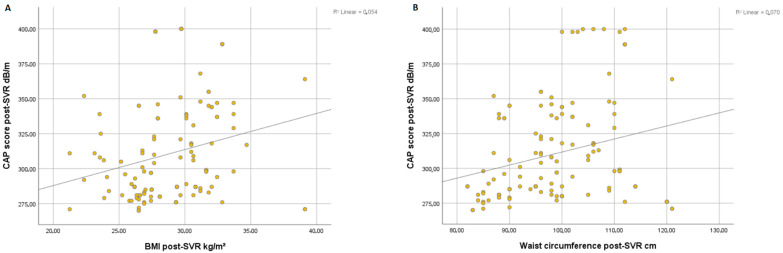
Correlation between CAP score and post-SVR and BMI (**A**) and waist circumference (**B**).

**Figure 2:**
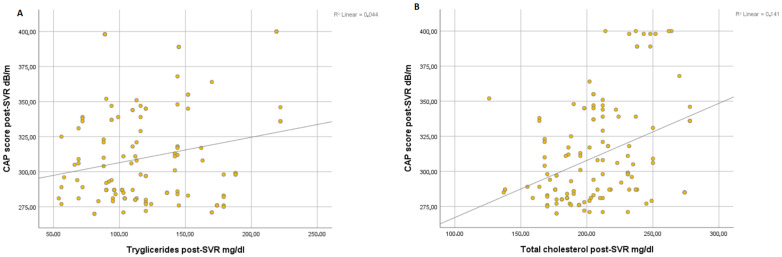
Correlation between CAP score and post-SVR and triglycerides (**A**) and total cholesterol (**B**).

Overall, we found a significant correlation between the degree of steatosis and clinical parameters of metabolic syndrome such as BMI (r = 0.054, *p* < 0.001) (Figure 1A) and WC (r = 0.070, *p* < 0.001) (Figure 1B) at post-SVR assessment. In addition, we observed a significant correlation between CAP score and levels of triglycerides at post-SVR evaluation (r = 0.044, *p* < 0.001) (Figure 2A) and total cholesterol values at post-SVR evaluation (r = 0.141, *p* < 0.001) (Figure 2B).

## 4. Discussion

Weight gain after SVR can have serious consequences in aggravating liver fibrosis. DAAs have been associated in studies with an increase in BMI among patients who have obtained SVR [21,22]. The direct causes are not fully known. It is assumed that it may be the consequence of metabolic disorders suffered during treatment or that a better quality of life after viral eradication may contribute to a less restrictive diet and, implicitly, to weight gain. In their research, Shousha et al. tried to find a correlation between antiviral treatment and an increase in BMI after achieving SVR. The results showed an increase in BMI in approximately 60% of patients who obtained SVR [21].

Schlevogt et al. evaluated the BMI in 264 patients with HCV cirrhosis who received interferon-free antiviral treatment for 12 weeks. Significant changes in BMI occurred in the entire group, with an observed increase in BMI by 35% compared to baseline at 12 weeks after the end of therapy and up to 44% at 24–48 weeks of follow-up [8]. Similar results are observed in our study, represented by an increase in BMI in patients that achieved SVR seen at 12 weeks after treatment and at more than 6 months in the post-SVR surveillance period.

A metabolism disruption in HCV-infected patients is encountered due to direct viral protein suppression. However, it is well-acknowledged that the HCV assembly and replication cycles are involved components of lipid metabolism, especially LDL which interact with viral proteins developing lipoviral particles and leading to a facilitating pathway for the entry into the hepatocytes process [22,23]. Therefore, the lipogenesis process is increased in HCV patients with a reduction in total cholesterol, HDL, and serum LDL-C levels [24]. Once the SVR is obtained, a reversal effect with a cancellation of the viral suppression is met with a rebound effect of serum lipid levels which leads to an increase in serum LDL-cholesterol and HDL-cholesterol levels after HCV clearance [25]. During the HCV treatment, significant changes in the LDL-cholesterol parameter with an increased level from both IFN-based regimens [4,26] and DAAs therapy [27,28] were observed, showing a direct impact of viral suppression on lipid metabolism [5].

Our data is similar to those reported in a recent study by Graf C et al. which concluded that is a significant increase in total cholesterol (158 ± 42.2 vs. 182.6 ± 37.7), LDL-cholesterol (94.8 ± 34.2 vs. 120.5 ± 39.8) and HDL-cholesterol (49.8 ± 12.7 vs. 53.8 ± 12.4) levels at 48 weeks after DAAs initiation therapy, while triglycerides levels appeared to be lower in comparison with our study [24].

Our study aims to increase awareness of the prevalence of fatty liver post-SVR and the necessity of screening and long-term follow-up. Additionally, we used VCTE, which is popular, simple, highly sensitive, and specific. Although liver biopsy is still the gold standard for fatty liver assessment and staging with MRI proton, density fat fraction may be more accurate; a liver biopsy is an invasive and expensive procedure, and many patients are reluctant to have it done due to concerns about pain and potential complications, even though they are rare [29]. The risk of inter- and intra-observer variability as well as sample error exists with biopsy. MRI procedures are relatively pricey. In post-SVR patients with normal liver enzymes, neither of these procedures is likely to be carried out. Therefore, using TE with CAP in a real-world scenario is plausible [30].

In a recent study, it was found that liver stiffness significantly lowers by about 3.1 kPa in the 6–12 months after viral eradication. Hepatic stiffness, however, does not diminish in patients who do not reach SVR [31]. An early drop is likely mostly attributable to the resolution of inflammation, whereas a decline that persists more than a year following EOT may be related to the regression of fibrosis, as has been seen in trials that matched liver biopsy with interferon-based therapy. The resolution of hepatic inflammation and the reversal of fibrosis are also likely contributing factors to the decrease in liver stiffness that occurs after viral eradication [32]. The results of our study suggest that mean LSM values had decreased after DAAs regimens, with an increasing CAP score, similar to another study of ours, which has been published recently [33]. Moreover, the high value of the CAP score at SVR post-evaluation is correlated with increasing BMI, WC, triglycerides, and total cholesterol levels.

Similar results were shown by Meissner et al. in 2016, who found an increase in LDL-cholesterol levels and a decrease in triglycerides levels at the time of SVR and post-SVR evaluation at 36 weeks and 48 weeks; however, compared to our study, they don’t describe significant changes in total cholesterol and HDL-cholesterol [5]. Furthermore, these kinds of changes in lipid metabolism were reported by other recent studies where an increase in total cholesterol and LDL-cholesterol were found; however, the authors couldn’t find significant changes in triglyceride levels [34,35]. In a similar study which involved a reduced number of patients (97 individuals with HCV), Estefan et al. found a significant increase in the level of triglycerides that appeared at the post-SVR evaluation (between 12 to 52 weeks) of the metabolic profile [36].

Instead, in the IFN-based regimens era, some different changes in lipid metabolism parameters were observed. In a study where were evaluated 330 patients were treated with Peg-INF/RBV, Ramcharran et al. reported an increased level of triglycerides and decreased levels of LDL-cholesterol and total cholesterol during the first 24 weeks of treatment [37]. In another study that assessed patients treated with Peg-IFN/RBV, genotype 1, the authors described a significant decrease in total cholesterol and triglyceride levels in patients who achieved SVR [38]. However, these lipid metabolism changes were correlated to higher rates of SVR in patients treated with IFN-based regimens. Harrison et al., in a study that was published in 2013, found that patients with higher levels of LDL-cholesterol had higher rates of SVR compared with those who had low levels of LDL-cholesterol [39].

In our study, we found important changes in lipid profile from the baseline to the post-SVR evaluation at 6 to 12 months. A significant increase in LDL-cholesterol and total cholesterol levels were achieved at the time of SVR and were maintained during the post-SVR follow-up period. Instead, the triglyceride and HDL-cholesterol levels presented high levels at the time of SVR and lower levels at post-SVR evaluation, which can be considered similar to baseline values. Patients receiving the DAA regimen can achieve SVR rates that are close to 99%, which is equivalent to the outcomes seen in controlled clinical studies. However, despite the elimination of the virus, Loo et al. found that over two-thirds of patients still required long-term follow-up due to severe liver disease [40]. These results suggest the presence of further underlying chronic liver diseases, such as NAFLD or alcoholic liver disease, which might accelerate the evolution of fibrosis and raise the chance of developing hepatocellular carcinoma. Therefore, to establish the prognosis and treatment options for individuals with chronic HCV infection after attaining SVR12, ongoing research and long-term follow-up studies are crucial.

Patients with HCV liver cirrhosis experience numerous metabolic changes such as an alteration of glycogen reserves; acceleration of lipolysis and the onset of malnutrition can be observed. Hypocholesterolemia and hypobetalipoproteinemia seem to dominate the biochemical picture in the case of HCV liver cirrhosis. The processes of synthesis, storage, and degradation of cholesterol and lipoproteins take place in the liver so that the changes in the liver parenchyma determined by the process of fibrogenesis and the onset of liver cirrhosis produce important changes. In liver cirrhosis, a disturbance of the esterification process is observed by decreasing the production of cholesterol and acyltransferase [41]. If the lipid synthesis chain is interrupted, the entire synthesis process is disrupted; therefore, a decrease in the LDL fraction is also observed. The decrease in very low-density lipoprotein (VLDL) levels is caused by the deficiency of a triglyceride transfer protein at the microsomal level. Apolipoprotein AI has a fundamental role in lipid metabolism, being directly correlated with the level of HDL at the serum level, the serum deficiency being considered a positive predictor for liver dysfunction [42]. This correlation between changes in lipid metabolism and the severity of liver cirrhosis assessed by the Child–Pugh prognostic score was carried out in a current research study by Bassani et al. The study showed that the level of total cholesterol is influenced by the stage of cirrhosis. Patients with Child–Pugh A score had higher circulating VLDL values compared to Child–Pugh B and Child–Pugh C. Lower values of total cholesterol, LDL, and HDL were recorded as being more frequent among patients with liver cirrhosis compared to patients diagnosed with hepatitis stage [41].

The cycle of viral replication and the mode of infection of the hepatocyte by HCV provided the perfect model for understanding the involvement of lipoproteins in favoring the spread of viral infection at the level of the hepatocyte. It is known that HCV uses receptors for LDL to promote entry into the hepatocyte, a mechanism that may undergo changes in the presence of antiviral treatment. To investigate whether direct antivirals can influence lipid fractions, Hashimoto et al. evaluated 100 patients treated with two regimens of direct antivirals and tried to understand whether the lipid profile undergoes an improvement under the action of the antiviral treatment, the lipid profile of the patients was evaluated 28 days after the initiation of therapy. During antiviral therapy, serum LDL and triglycerides have been found to increase compared to baseline values. It can be assumed in this case that different regimens of direct antivirals contribute, depending on their mechanism of action, to the modification of the lipid profile. Patients treated with Sofosbuvir, a polymerase inhibitor, had higher values compared to those treated with Daclatasvir, an NS5A inhibitor, from which we can conclude that these changes in serum lipids are not permanent, being directly influenced by the pharmacokinetics of DAAs-regimen [43].

Our study’s assessment of a comprehensive metabolic profile, which is known to contribute to fat accumulation in the liver, particularly in people with HCV infection, is one of its strengths. The first drawback is that, in our investigation, the median follow-up period was a maximum of 52 weeks following the end of the course of treatment.

The measures of LSM and CAP at post-SVR evaluation, without any further follow-up, reflect another restriction. Longer investigations are required to monitor the development of liver steatosis and fibrosis in HCV-infected patients treated with DAAs regimens [44]. One drawback that needs to be noted is the lack of histological evaluation. Finally, we did not collect information about the patient’s eating habits, physical activity, or lifestyle data.

## 5. Conclusions

Although interferon-free treatment has proven a high rate of obtaining SVR in our patients, it has been associated with weight gain, increased WC, and lipid metabolism alterations in the long-term follow-up after HCV clearance. Moreover, the increasing degree of steatosis is associated with raised components of the metabolic profile identified at post-SVR evaluation. Acknowledgment of the precise mechanism and the subtype of patients that acquire modifications of metabolic syndrome components are essential because they may erase the beneficial effects of the HCV cure due to the worsening of hepatic fibrosis.

## Figures and Tables

**Table 1:** Baseline characteristics of the study population.

Variables	Overall Cohort *n*, 132
Age (years)	61.17 ± 9.11
Females, *n* (%)	85 (64.4)
Weight(kg)	79.12 ± 16.03
Height(cm)	168.5 ± 9.69
Body mass index (kg/m^2^)	27.12 ± 3.22
Underweight, *n* (%)	3 (2.3)
Lean subjects, *n* (%)	42 (31.8)
Overweight, *n* (%)	53 (40.2)
Obese, *n* (%)	34 (25.7)
Waist circumference, (cm)	87.6 ± 14.1
Hypertension, *n* (%)	68 (51.5)
Diabetes, *n* (%)	32 (24.2)
Pre-diabetes, *n* (%)	39 (32.5)
Dyslipidemia, *n* (%)	27 (20.4)
Metabolic syndrome, *n* (%)	35 (26.5)
Steatosis degree, *n* (%)	
CAP < 248 dB/m, *n* (%)	57 (43.2)
CAP ≥ 248 dB/m, *n*(%)	32 (24.2)
CAP ≥ 268 dB/m, *n* (%)	26 (19.7)
CAP ≥ 280 dB/m, *n* (%)	17 (12.9)
CAP, dB/m	233.27 ± 48.26
Fibrosis stage, *n* (%)	
LSM < 5.6 kPa, *n* (%)	18 (13.6)
LSM ≥ 5.6 kPa, *n* (%)	37 (28)
LSM ≥ 7.1 kPa, *n* (%)	16 (12.1)
LSM ≥ 9.5 kPa, *n* (%)	42 (31.8)
LSM ≥ 12.5 kPa, *n* (%)	19 (14.4)
LSM, kPa	8.94 ± 5.1

*n*—number of subjects; CAP, controlled attenuation parameter; LSM, liver stiffness measurement.

**Table 2:** Patients laboratory data pre- and post-DAAs regimens.

Variable	Pre-Treatment	Post-Treatment	*p*-Value
HGB (g/dL)	12.91 *±* 1.72	12.94 *±* 1.58	0.644
Platelet count (G/L)	176.73 *±* 68.06	183.53 *±* 66.43	0.721
Total bilirubin (mg/dL)	1.19 *±* 0.64	1.08 *±* 0.55	0.532
AST (IU/L)	36.83 ± 21.54	30.62 *±* 23.61	0.097
ALT (IU/L)	42.5 ± 25.21	35.23 *±* 22.13	0.119
GGT (IU/L)	90.4 ± 72.5	62.4 ± 46.3	0.038
Albumin(mg/dL)	3.86 *±* 0.72	4.01 ±0.67	0.058
Glucose(mg/dL)	115.08 ± 34.9	109.26 *±* 29.43	0.064
HbA1c (%)	5.52 ± 0.65	5.41 ± 0.51	0.124
Total cholesterol (mg/dL)	161.68 ± 31.6	177.01 ± 42.2	0.014
Triglycerides (mg/dL)	135.7 ± 41.4	145.4± 47.2	0.026
LDL (mg/dL)	114.05± 37.1	129. 32 ± 44.6	0.017
HDL (mg/dL)	44.8 ± 12.9	43.95 ± 13.1	0.236
LSM (kPa)	8.94 ± 5.1	8.02 ± 4.8	0.023
Fibrosis stage, *n* (%)			
F0, *n* (%)	18 (13.6)	25 (18.9)	
F1, *n* (%)	37 (28)	42 (31.8)	
F2, *n* (%)	16 (12.1)	19 (14.4)	
F3, *n* (%)	42 (31.8)	34 (25.8)	
F4, *n* (%)	19 (14.4)	12 (9.1)	
CAP (dB/m)	233.27 ± 48.26	259 ± 63.89	<0.001
Steatosis degree, *n* (%)			
S0, *n* (%)	57 (43.2)	50 (37.9)	
S1, *n* (%)	32 (24.2)	39 (29.5)	
S2, *n* (%)	26 (19.7)	29 (22)	
S3, *n* (%)	17 (12.9)	14 (10.6)	

HGB, total hemoglobin; AST, aspartate aminotransferase; ALT, alanine aminotransferase; GGT, gamma-glutamyl transferase; HbA1C, glycosilated hemoglobin, LDL, low-density lipoprotein cholesterol; HDL, high-density lipoprotein cholesterol; CAP, controlled attenuation parameter; LSM, liver stiffness measurements.

**Table 3:** Evolution of clinical and metabolic profile during DAAs regimen.

Variable	Baseline	SVR12	Post-SVR Evaluation	*p*-Value
BMI (kg/m^2^)	27.11 ± 3.22	27.415 ± 3.03	28.04 ± 1.11	0.012
Waist circumference (cm)	87.6 ± 13.1	87.9 ± 13.2	88.4 ± 13.6	0.031
Cholesterol (mg/dL)	161.68 ± 31.6	168.23 ± 37.1	177.01 ± 42.2	0.014
LDL-cholesterol (mg/dL)	114.05± 37.1	122.41 ± 34.3	129. 32 ± 44.6	0.07
Triglycerides (mg/dL)	135.7 ± 41.4	133.48 ± 41.8	145.4 ± 47.2	0.026

BMI, body max index; LDL-cholesterol, low-density lipoprotein cholesterol.

## Data Availability

The corresponding author can provide the data described in this study upon request. Since the data are the property of the Institute of Gastroenterology and Hepatology in Iasi, Romania, they are not accessible to the general public.
